# Supplementary material for: Empirical methods for controlling false positives and estimating confidence in ChIP-Seq peaks
Source: BMC Bioinformatics. 2008 Dec 5;9:523. doi: 10.1186/1471-2105-9-523 (PMC2628906; doi:10.1186/1471-2105-9-523)
Supplement: Additional File 1 — A variety of html documents from the USeq web site detailing the available applications, their best usage, output file type descriptions, command line menus, etc. [file 1471-2105-9-523-S1.zip › USeqUserGuides/JavaDoc.zip!/JavaDoc/util/bio/annotation/Translation.html]

Translation


|  |  |  |
| --- | --- | --- |
| **Overview** \| **Package** \| **Class** \| **Use** \| **Tree** \| **Deprecated** \| **Index** \| **Help** | |  |
| **PREV CLASS**   NEXT CLASS | **FRAMES**    **NO FRAMES**     **All Classes** |  |
| SUMMARY: NESTED \| FIELD \| CONSTR \| METHOD | DETAIL: FIELD \| CONSTR \| METHOD |  |


---


## util.bio.annotation Class Translation

```
java.lang.Object
  util.bio.annotation.Translation
```

**All Implemented Interfaces:**: java.io.Serializable

---

``` public class Translation extends java.lang.Object implements java.io.Serializable ```

Translation specific information, protein but referenced with genomic coordinates thus some
bits code for protein, some don't physically exist.

**See Also:**: Serialized Form

---

**Constructor Summary**

`Translation(Gff3Feature f)`

`Translation(int start,
int end,
int orientation)`


| **Method Summary** | |
| --- | --- |
| `java.util.ArrayList` | `getCodingSegments()` |
| `int` | `getEnd()` |
| `java.util.ArrayList` | `getIntrons()` |
| `int` | `getOrientation()` |
| `int` | `getStart()` |
| `void` | `makeSegments()` |
| `void` | `setTransGrp(TransGroup ref)`             Sets TransGroup ref but also fires the makeSegments method |
| `java.lang.String` | `toString()` |
| `static java.lang.String` | `toStringALInts(java.util.ArrayList al)` |

**Methods inherited from class java.lang.Object**

`equals, getClass, hashCode, notify, notifyAll, wait, wait, wait`

**Constructor Detail**

### Translation

```
public Translation(int start,
                   int end,
                   int orientation)
```

---


### Translation

```
public Translation(Gff3Feature f)
```


**Method Detail**

### setTransGrp

```
public void setTransGrp(TransGroup ref)
```

:   Sets TransGroup ref but also fires the makeSegments method

---


### makeSegments

```
public void makeSegments()
```

---


### toString

```
public java.lang.String toString()
```

:   **Overrides:**: `toString` in class `java.lang.Object`

---


### toStringALInts

```
public static java.lang.String toStringALInts(java.util.ArrayList al)
```

---


### getCodingSegments

```
public java.util.ArrayList getCodingSegments()
```

---


### getOrientation

```
public int getOrientation()
```

---


### getEnd

```
public int getEnd()
```

---


### getStart

```
public int getStart()
```

---


### getIntrons

```
public java.util.ArrayList getIntrons()
```


---


|  |  |  |
| --- | --- | --- |
| **Overview** \| **Package** \| **Class** \| **Use** \| **Tree** \| **Deprecated** \| **Index** \| **Help** | |  |
| **PREV CLASS**   NEXT CLASS | **FRAMES**    **NO FRAMES**     **All Classes** |  |
| SUMMARY: NESTED \| FIELD \| CONSTR \| METHOD | DETAIL: FIELD \| CONSTR \| METHOD |  |


---
